# Native and Introduced Plants in a Managed Campus Landscape: Habitat Associations, Functional Overlap, and Taxonomic Patterns

**DOI:** 10.1002/ece3.74348

**Published:** 2026-09-17

**Authors:** Santosh K. Rana, Gary L. Davenport, Serene Awad, Donald J. Webb, Samuel G. Esposito, Cuong D. Nguyen, Ian J. Renne

**Affiliations:** ^1^ Department of Chemical and Biological Sciences Youngstown State University Youngstown Ohio USA

**Keywords:** floristic composition, habitat association, managed landscape, native and introduced species, plant community assembly, taxonomic structure

## Abstract

Managed landscapes support diverse vascular plant assemblages, with composition shaped by both regional species pools and human planting and maintenance. This study assessed floristic composition, habitat associations, growth forms, and taxonomic structure of vascular plants across managed landscapes on the Youngstown State University (YSU) campus, Ohio. In total, 112 species from 56 families were recorded, comprising 77 introduced and 35 native taxa. Introduced species were disproportionately associated with managed habitats, particularly flowerbeds and beds, while native species occurred more frequently than expected in lawns. Life‐form composition also differed between nativity groups, with native assemblages dominated by woody species (~63%) and introduced assemblages enriched in herbaceous and shrubby taxa (~61%). Trait‐based clustering revealed substantial overlap between native and introduced species in functional‐taxonomic space, although introduced taxa were concentrated within a limited number of species‐rich families. These findings demonstrate clear association among species origin, habitat type, growth form, and taxonomic composition within this managed campus landscape. The observed patterns suggest a strong influence of horticultural selection and landscape management on campus floristic composition. However, the observational, single‐season design does not allow for the distinction of demographic processes, propagule pressure, competitive interactions, or invasion dynamics. Managed campuses therefore provide valuable case‐study systems for documenting the distribution of native and introduced floras under intensive human management and for developing hypotheses that can be tested through longitudinal and demographic research.

## Graphical abstract

This study examined the distribution, habitat associations, and functional and taxonomic composition of native and introduced plant species across a highly managed university campus landscape. Native and introduced taxa differed in their associations with major habitat types and in growth‐form and taxonomic composition, while substantial functional–taxonomic overlap occurred between the two groups. These findings highlight how sustained landscape management and ecological characteristics jointly structure native and introduced plant assemblages in managed urban environments.

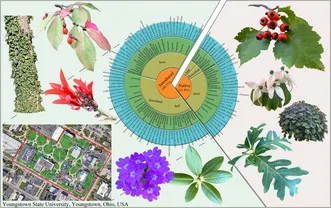

## 1 Introduction

Vascular plant assemblages in managed landscapes are shaped by interactions between ecological processes and sustained human intervention. As vegetated environments such as parks, streetscapes, residential yards, and institutional campuses expand globally, they now comprise a substantial proportion of managed land, often exceeding the spatial extent of nearby natural remnants (McKinney 2002; Aronson et al. 2014). These systems provide opportunities for plant persistence and ecological interactions, yet their composition is largely governed by planting decisions, maintenance regimes, and landscape design. Consequently, plant communities in these settings are assembled through both natural ecological filtering and deliberate management, resulting in floras that often diverge from regional species pools in both functional and taxonomic structure (Martin et al. 2009; Pickett et al. 2011; Kendal et al. 2012; Miguez et al. 2025).

A defining characteristic of managed plant assemblages is the widespread occurrence of introduced (non‐native) species, particularly in highly visible and intensively maintained habitats. Introduced ornamentals are commonly selected for traits such as rapid establishment, uniform growth, and tolerance to disturbance, which align with horticultural objectives (Kermath 2007; Kendal et al. 2012). In contrast, native plants are increasingly recognized for their ecological importance, including support for native fauna, contributions to ecosystem stability, and representation of regional biogeographic history (Tallamy 2007; Berthon et al. 2021; Russo et al. 2025; Souther et al. 2026). The prevalence of introduced species in public landscapes may arise through multiple, non‐exclusive processes, including deliberate horticultural selection, repeated planting, environmental filtering, naturalization, and, for a subset of taxa, subsequent invasion. Distinguishing among these processes requires demographic and temporal information, but floristic patterns can reveal how native and introduced taxa differ in their associations with managed habitats, functional attributes, and taxonomic composition.

Much of the scientific discourse on introduced plants has focused on the factors driving biological invasions and their ecological consequences (Davis et al. 2005; Renne et al. 2006; Aslan 2019). Large‐scale analyses show that non‐native species can reduce native species richness, particularly when functionally and phylogenetically similar to resident taxa (Conti et al. 2018; Guo et al. 2021; Liu et al. 2025). However, these effects are strongly context‐dependent and influenced by spatial scale, disturbance regimes, and management intensity (Renne and Tracy 2003; Stohlgren et al. 2003; Fridley et al. 2007; USGS 2023). In managed landscapes, many introduced species are deliberately planted and maintained through horticultural practices, and the horticultural trade represents a major pathway of global plant introduction (Mack and Lonsdale 2001; Martin et al. 2009; Hulme et al. 2018). Introduced status, however, does not imply invasiveness. Non‐native plants may occur along a continuum from introduced or occasional taxa to naturalized populations and, for a smaller subset, invasive species that spread beyond sites of introduction (Richardson et al. 2000; Blackburn et al. 2011). Distinguishing among these stages requires evidence of establishment, reproduction, persistence, and spread that occurrence alone cannot provide. Horticultural selection, repeated introduction, environmental conditions, establishment, and subsequent spread may operate sequentially and interactively; thus, management represents one component of the broader introduction–naturalization–invasion process rather than an alternative to it. Accordingly, we use “introduced” for non‐native taxa recorded on campus and do not assume that all introduced taxa are invasive.

A functional‐trait perspective provides a useful framework for examining compositional similarities and differences between native and introduced plants. Introduced species in managed landscapes may possess trait combinations favored by horticultural selection and maintenance, while native taxa may share only some of these attributes. Comparing their functional composition can therefore identify patterns that generate hypotheses about the processes shaping managed floras (Pyšek et al. 2008; Cavender‐Bares et al. 2018). Such comparisons are particularly informative in public landscapes, where planting regimes, maintenance practices, and esthetic priorities may influence which species are represented. However, distinguishing horticultural selection from ecological filtering or differential performance requires additional demographic, environmental, or experimental data.

College campuses offer an ideal but underutilized system for addressing this knowledge gap. Campuses typically encompass a mosaic of habitats, from curated ornamental beds to lawns and semi‐natural green spaces, within a compact and spatially structured landscape. Their standardized management practices and long‐term institutional stewardship make campuses valuable model systems for examining how landscape design and maintenance shape plant community assembly (Kermath 2007; Kendal et al. 2017). Youngstown State University (YSU), located in northeastern Ohio, exemplifies this context, combining extensive green space with a long‐standing commitment to campus forestry yet lacking a comprehensive assessment of floristic structure. Using the Youngstown State University campus as a case study, we addressed three questions: (1) how are native and introduced species distributed among lawns, beds, and flowerbeds; (2) how do native and introduced assemblages differ in growth form and taxonomic composition; and (3) to what extent do the native and introduced species overlap in functional–taxonomic space? By integrating habitat associations, growth forms, and taxonomic structure, we characterize patterns of native and introduced plant assembly within a managed landscape and evaluate whether these patterns are consistent with horticultural and management‐associated filtering. Because our study is based on a single‐season floristic survey, we treat these patterns as associations rather than direct evidence of demographic, competitive, or invasion mechanisms.

## 2 Materials and Methods

### 2.1 Study Area

We conducted this study on the southeastern portion of the Youngstown State University (YSU) campus in Youngstown, Ohio, USA, which has a median elevation of 279 m above sea level. The surveyed area covers approximately 6.7 ha with a perimeter of ~1180 m. The site occurs in an urban matrix and consists of a mosaic of managed lawns, ornamental beds, and flowerbeds typical of public institutional landscapes in the northeastern United States. Such campuses represent increasingly important but understudied urban ecosystems where landscaping and maintenance decisions exert strong ecological filters on plant community composition (McKinney 2002; Pickett et al. 2011; Berthon et al. 2021).

### 2.2 Floristic Survey and Data Collection

Floristic surveys were conducted before the first frost of the autumn season (2nd and 23rd October 2025), ensuring that most species retained identifiable vegetative or reproductive structures, using a systematic walk‐through approach commonly applied in urban vegetation assessments (Aronson et al. 2014; Kendal et al. 2017). Surveyed areas were divided among three field teams that recorded all vascular plant species encountered within lawns and landscaped planting areas. Species locations were recorded using adjacent buildings, walkways, and roads as spatial reference points rather than fixed plots, reflecting the heterogeneous and design‐driven nature of campus landscapes.

For each species occurrence, three digital photographs were taken to capture (i) whole‐plant architecture, (ii) vegetative morphology, and (iii) reproductive structures when present. This approach allowed for post hoc species verification and reduced identification uncertainty associated with seasonal phenology, particularly given autumn sampling (Bonney et al. 2014).

### 2.3 Species Identification and Classification

Species identification was conducted using regional floras and field guides (Gleason and Cronquist 1991), supplemented by expert consultation and verification using iNaturalist, which has been shown to improve accuracy and reproducibility in floristic surveys when combined with expert validation (Bonney et al. 2014). Nomenclature and family assignments followed APG IV (Angiosperm Phylogeny Group et al. 2016).

Each recorded species was classified according to:

1. Nativity status (native or introduced),
2. Growth form (tree, shrub, vine, herbaceous perennial, herbaceous annual),
3. Taxonomic family, and
4. Habitat type (lawn, bed, flowerbed).

We determined nativity status using distribution data from the Biota of North America Program, a widely used reference for continental‐scale plant distributions (Kartesz 2015). Introduced species were additionally cross‐referenced against the Ohio Invasive Plants Council (OIPC; Ohio Invasive Plants Council 2025) list to determine whether they had been assessed as invasive or potentially invasive in Ohio. We recorded cultivars at the species level when we could not reliably distinguish precise cultivar identity in the field, a common limitation in urban floristic surveys (Kendal et al. 2012).

Habitat categories represent broad environmental and land‐use contexts and were not intended as quantitative proxies for management or disturbance intensity. Although beds, flowerbeds, and lawns differ in their typical planting and maintenance practices, we did not directly quantify management variables such as mowing frequency, irrigation, fertilizer application, planting frequency, or plant replacement. Accordingly, we interpreted habitat associations as compositional patterns associated with landscape type rather than as direct estimates of management effects.

### 2.4 Data Structure and Analytical Units

To avoid pseudo‐replication and over‐weighting of common planted or naturally occurring species, we conducted analyses using unique species × habitat × origin records rather than abundance or frequency counts. This conservative approach emphasizes community composition and trait structure over planting density and aligns with community‐level analyses in urban ecology and invasion research (Aronson et al. 2016; Pyšek et al. 2008). We performed all data cleaning, standardization, and factor harmonization in R v4.4.0 (R Core Team 2024), including removing incomplete records and consistently releveling categorical variables to ensure comparability across analyses.

### 2.5 Visualization of Floristic Structure

To characterize differences between native and introduced species across functional, spatial, and taxonomic dimensions, we generated complementary visualizations. Data manipulation and plotting were conducted in R v4.4.0 (R Core Team 2024) using the packages *dplyr* v1.1.4 (Wickham et al. 2025), *tidyr* v1.3.1 (Wickham et al. 2024), and *ggplot2* v3.5.1 (Wickham 2016). We visualized hierarchical relationships among origin, habitat type, family, and species using sunburst and alluvial (Sankey) diagrams implemented with the package *ggalluvial* v0.12.5 (Brunson 2020), with ribbon widths proportional to species richness. Such flow‐based visualizations are increasingly used to summarize complex categorical structure in ecological datasets (Rosvall and Bergstrom 2010). We summarized functional composition by calculating the proportional representation of major growth forms within native and introduced assemblages and visualized it using radial (polar) bar plots, which effectively highlight contrasts in functional dominance and trait skew (Wegman 2000).

### 2.6 Trait‐Based Clustering and Dissimilarity Analysis

To examine whether native and introduced species occupy distinct or overlapping functional and taxonomic space, we conducted hierarchical clustering based on mixed categorical traits, including growth form, taxonomic family, habitat type, and origin. We calculated pairwise dissimilarities using Gower distance, which accommodates mixed data types (Gower 1971). We clustered species using Ward's minimum variance method, which minimizes within‐cluster variance and is widely applied in ecological trait analyses (Ward 1963; Legendre and Legendre 2012). We used the resulting dendrograms to assess patterns of trait separation between native and introduced taxa.

### 2.7 Statistical Analysis

We tested associations between species origin and habitat type using Pearson's chi‐square tests, appropriate for categorical community‐level data (Agresti 2019). Effect sizes were quantified using Cramer's V, allowing assessment of association strength independent of sample size (Cramér 1999). To interpret habitat‐specific deviations from expected distributions, we calculated preference indices (observed/expected ratios) and standardized residuals, which identify over‐ and under‐representation of species origins within habitats (Manly et al. 2002; Sharpe 2015). We compared mean species richness per habitat for native and introduced species using paired *t*‐tests, treating habitats as paired observational units. Given the small number of habitat categories, we corroborated results using Wilcoxon signed‐rank tests as a non‐parametric alternative (Conover 1999). Finally, we modeled the relationship between introduced species dominance and habitat type using logistic regression, treating habitat category (bed, lawn, flowerbed) as a categorical predictor. Beds are mixed ornamental plantings containing shrubs or perennial ornamentals, flowerbeds are intensively managed ornamental plantings dominated by seasonal flowers, and lawns are turfgrass‐dominated areas maintained primarily through mowing (Hosmer et al. 2013).

## 3 Results

### 3.1 Floristic Composition and Habitat Distribution

**FIGURE 1:**
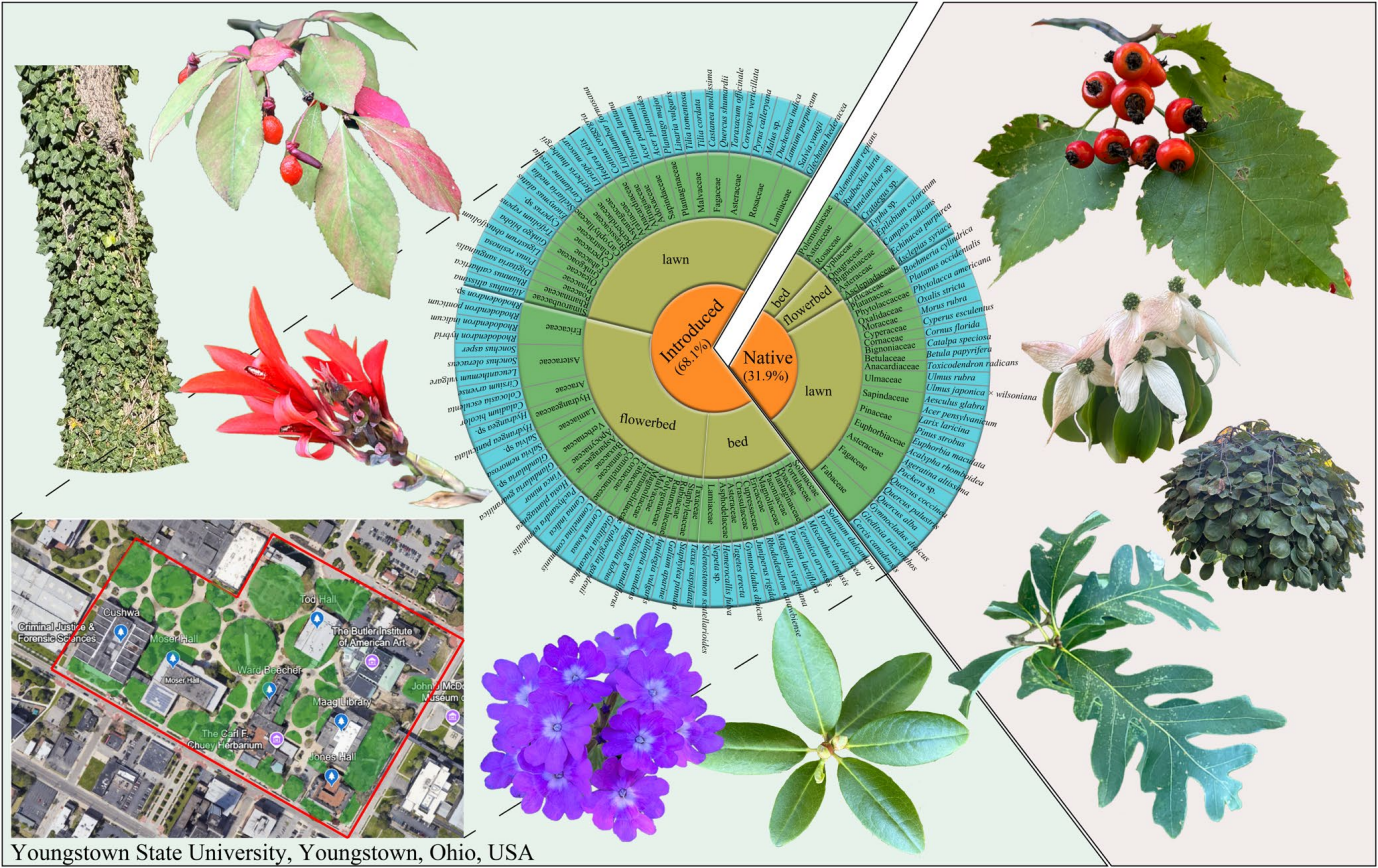
Hierarchical structure of campus flora at Youngstown State University (Ohio, USA). Sunburst diagram illustrating the hierarchical organization of plant species by origin (native vs. introduced), habitat type (bed, lawn, flowerbed), taxonomic family, and species across managed campus environments. Percentages in the central rings represent the relative contribution of native and introduced species to the total flora. The inset map (lower left) shows the sampled area on the Youngstown State University campus, with surveyed locations highlighted in transparent green. Species represented in the hierarchical structure correspond to the native and introduced taxa recorded within the sampled area (See Table S1). All plant photographs were captured by the authors within the study site.

**FIGURE 2:**
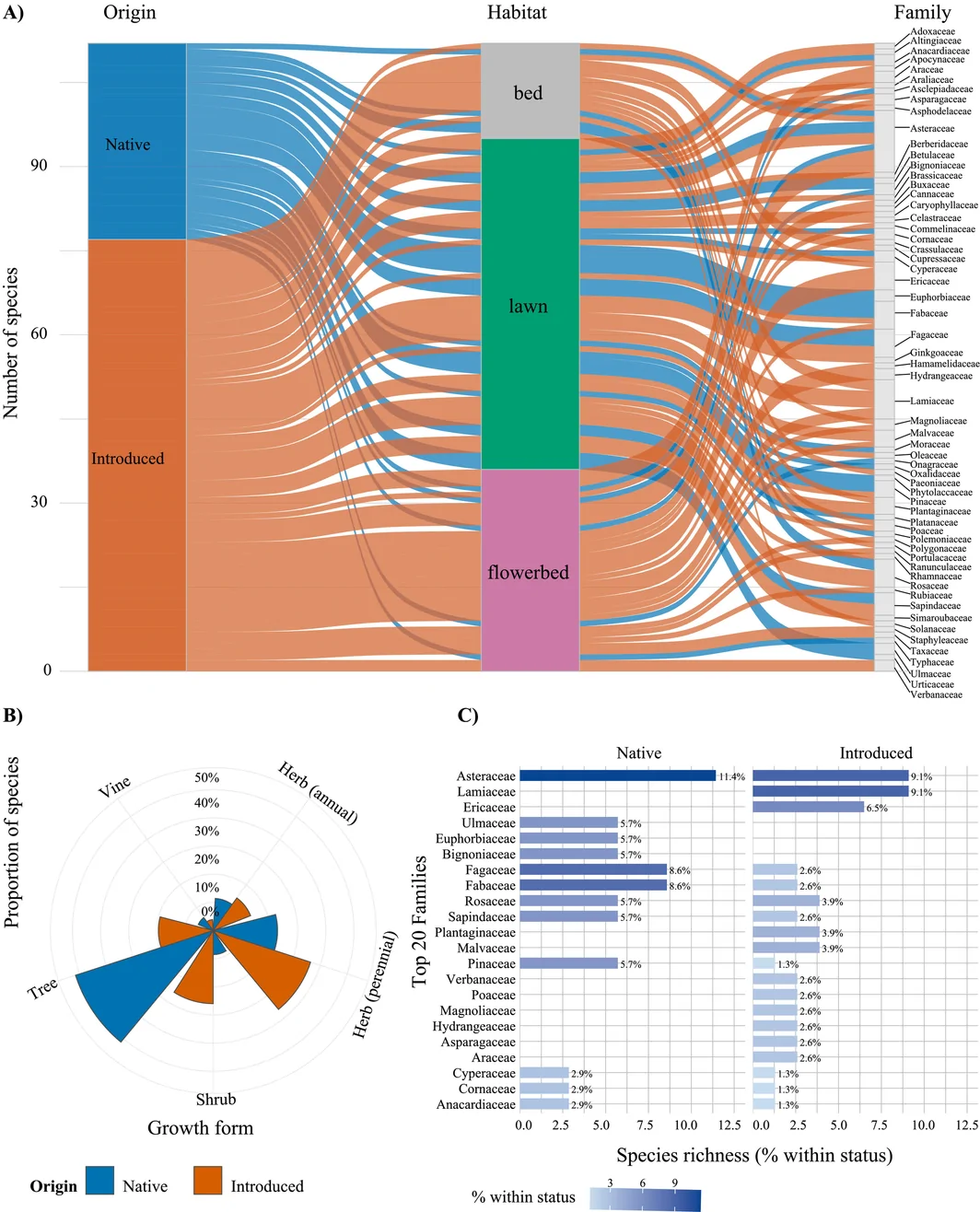
Taxonomic, functional, and habitat‐level structure of native and introduced plant species on campus. (A) Alluvial diagram showing species flows from origin (native vs. introduced) to habitat type (bed, lawn, flowerbed) and plant family, with ribbon widths proportional to species counts. (B) Radial plot illustrating proportional representation of major growth forms for native (blue) and introduced (orange) species. (C) Top 20 plant families ranked by total species richness (including tied ranks), expressed as the percentage of species within native and introduced groups. Bar lengths indicate relative contribution within each status, allowing comparison of dominant families between native and introduced floras.

Across surveyed areas of the YSU campus, we recorded 112 vascular plant species representing 56 families (Figures 1 and 2A and Table S1). Introduced species comprised ~68% of the flora, whereas native species accounted for ~32% (Table 1). Only a limited subset of introduced species was classified as invasive or potentially invasive by the Ohio Invasive Plants Council, indicating that most introduced taxa were not classified as invasive or potentially invasive by OIPC.

**TABLE 1:** Distribution of native and introduced plant species across habitat types and results of association tests.

Habitat type	Native species (*n*)	Introduced species (*n*)	Total (*n*)
Bed	4	13	17
Lawn	26	33	59
Flowerbed	5	31	36
Total	35	77	112
Pearson's *χ* ^2^ (df = 2)	10.03		
*p*‐value	0.0066		
Cramer's V	0.3		

*Note:* Observed frequencies of native and introduced plant species across three managed habitat types. Pearson's chi‐square test indicates a significant association between species origin and habitat type, with a moderate effect size based on Cramer's V.

Species were distributed across three habitat types, namely lawns, beds, and flowerbeds, that differed in design and management intensity. Lawns supported the highest floristic richness (59 species; 53% of the total flora), followed by flowerbeds (36 species; 32%) and beds (17 species; 15%), reflecting differences in spatial extent and planting density among habitat types (Table 1). These baseline patterns provide the context for subsequent analyses of habitat associations, functional traits, and taxonomic structure.

### 3.2 Functional Composition of Native and Introduced Assemblages

Life‐form distributions differed markedly between native and introduced assemblages (Figure 2B). Native species were dominated by long‐lived woody growth forms, particularly trees, which accounted for 63% of native species (22 of 35). In contrast, introduced species were enriched in herbaceous perennials and ornamental shrubs, which together comprised ~61% of introduced species (47 of 77). Herbaceous annuals and vines contributed < 10% of species richness within either group. These functional patterns were consistent across habitat types. Native woody species occurred most frequently in lawns, whereas herbaceous and shrubby introduced species were concentrated in beds and flowerbeds (Figure 2A). Thus, plant life‐form composition differed between native and introduced assemblages and among the habitat types.

### 3.3 Habitat Associations and Species Richness Patterns

**FIGURE 3:**
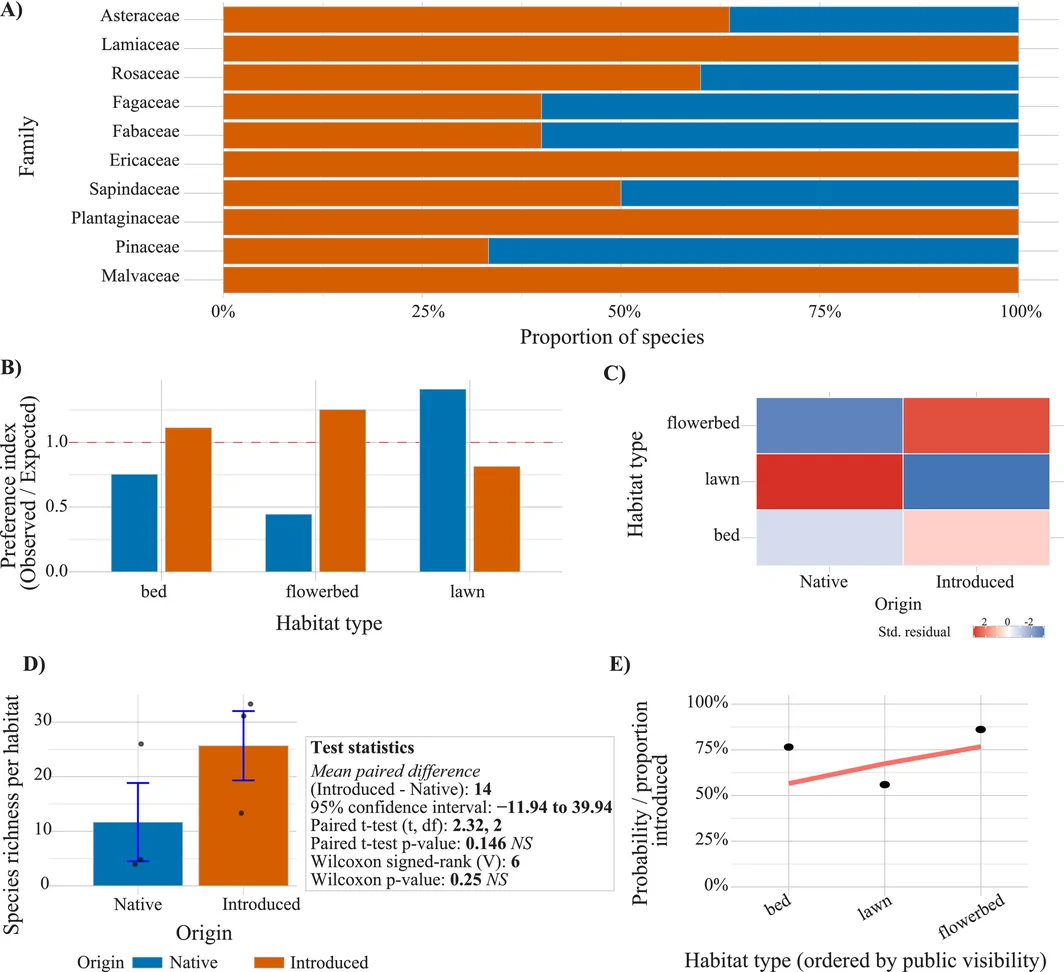
Habitat‐ and family‐level patterns distinguishing native and introduced plant species across campus environments. (A) Taxonomic (family‐level) bias in landscaping, showing the proportion of native (blue) and introduced (orange) species within dominant plant families. (B) Habitat‐specific preference index derived from Pearson's chi‐square expectations, calculated as the ratio of observed to expected species counts. Values > 1 indicate over‐representation of a species origin within a habitat. (C) Heatmap of standardized residuals from the Pearson chi‐square test assessing the association between species origin and habitat type. (D) Mean species richness per habitat for native and introduced species (±SE), with individual habitat values overlaid. Inset statistics summarize paired comparisons treating habitats as paired units for paired *t*‐test and Wilcoxon signed‐rank test. (E) Relationship between habitat type and the dominance of introduced species. Points represent observed proportions of introduced species within each habitat, and the line represents fitted probabilities from a logistic regression model.

Contingency analysis revealed a significant association between species origin and habitat type (Pearson's *χ* ^2^ = 10.03, df = 2, *p*‐value = 0.0066), with a moderate effect size (Cramer's *V* = 0.30; Table 1). Introduced species were numerically dominant in all habitats but were strongly over‐represented in flowerbeds (31 introduced vs. 5 native species) and beds (13 vs. 4 species), whereas native species occurred more frequently than expected in lawns (26 native vs. 33 introduced species; Table 1 and Figure 3B,C).

Habitat‐specific preference indices (observed/expected ratios under chi‐square expectations) further clarified these patterns. Introduced species exhibited preference index values > 1 in flowerbeds and beds, indicating over‐representation relative to random expectation, while native species showed preference index values > 1 primarily in lawns (Figure 3B). Standardized residuals from the chi‐square test corroborated these trends, with strong positive residuals for introduced species in flowerbeds and for native species in lawns (Figure 3C).

When species richness was summarized at the habitat level and compared between native and introduced taxa, introduced species had higher mean richness per habitat than native species (mean paired difference = 14 species; Figure 3D; inset text box). However, this difference was not statistically significant under either paired *t*‐tests (*p*‐value = 0.146) or Wilcoxon signed‐rank tests (*p*‐value = 0.25). Although statistical support was limited by the small number of habitat categories (*n* = 3), richness differences were directionally consistent across habitats, with introduced species exhibiting higher counts in beds, lawns, and flowerbeds (Figure 3D; inset text box).

Logistic regression modeling further showed that the proportion of introduced species varied systematically among habitat types (Figure 3E), decreasing from beds to lawns, and reaching its highest value in flowerbeds (Figure 3E). Observed proportions closely matched fitted probabilities from the logistic model, indicating a consistent gradient in introduced species prevalence across habitat types.

### 3.4 Taxonomic Structure and Trait Clustering

Taxonomic composition differed markedly between native and introduced assemblages. Introduced taxa were concentrated within a few species‐rich families, whereas native species were distributed across a broader range of lineages (Figure 1). Alluvial maps showed that introduced species were disproportionately associated with families commonly represented in horticulture, including Asteraceae, Lamiaceae, and Ericaceae, and were primarily linked to ornamental habitats (Figure 2A). In contrast, native species were more evenly distributed among families characteristic of regional temperate forest and meadow systems and occurred primarily in lawns (Figure 2A).

Family‐level rankings further highlighted this taxonomic bias, with dominant families contributing disproportionately to introduced assemblages relative to native ones (Figures 2C and 3A). Hierarchical clustering based on mixed traits (life form, family, habitat type, and origin) revealed both separation and overlap between native and introduced taxa (Figure S1). While several clusters were enriched for one origin group, many contained both native and introduced species, indicating substantial overlap in the measured functional–taxonomic attributes. Thus, native and introduced taxa showed compositional differentiation without complete separation in the multivariate space represented by the measured attributes.

## 4 Discussion

### 4.1 Habitat‐Associated Patterns of Native and Introduced Species in a Managed Landscape

Species origin was strongly associated with habitat type across the Youngstown State University campus. Introduced taxa were disproportionately represented in highly managed ornamental habitats, particularly flowerbeds, whereas native taxa were more frequently associated with lawns. Habitat‐specific preference indices and residual patterns further supported this differentiation. Together, these results show that native and introduced species are not randomly distributed among the major landscape types represented on campus.

The concentration of introduced taxa in beds and flowerbeds is consistent with the prominent role of human planting and landscape management in structuring urban and managed plant assemblages (Pickett et al. 2011; Aronson et al. 2016). Managed landscapes are intentionally assembled environments in which floristic composition can reflect planting decisions, maintenance practices, landscape design, and local ecological conditions (Pickett et al. 2011; Aronson et al. 2016). Horticulture also represents an important pathway for introducing non‐native plants into managed environments, and plant availability, propagation, and human preferences can influence which taxa are represented in urban floras (Russo et al. 2025). Thus, the observed association between introduced taxa and ornamental habitats is consistent with an important role for horticultural selection and management, but the present data do not identify the relative contribution of individual processes responsible for this pattern.

This distinction between habitat association and evidence of biological invasion is particularly important when interpreting these findings. The greater representation of introduced taxa in ornamental habitats does not, by itself, indicate that these species are invasive or causing ecological impacts. The consequences of introduced taxa vary among spatial scales, disturbance histories, and management contexts (USGS 2023). Likewise, introduced status alone does not demonstrate competitive superiority or displacement of native species. Our survey did not distinguish deliberately planted from self‐established individuals or quantify planting frequency, propagule pressure, recruitment, mortality, or temporal population change. Consequently, the prevalence of introduced taxa in ornamental habitats could reflect deliberate planting and maintenance, spontaneous establishment, naturalization, or combinations of these processes. Distinguishing among these possibilities will require demographic and temporal information beyond the scope of the present survey.

The comparatively greater representation of native taxa in lawns is nevertheless an important result. Although lawns themselves are managed environments, they contained a larger native component than the ornamental habitats examined here. Comparable studies have shown that urban green spaces can retain substantial components of regional native floras despite intensive surrounding development (Aronson et al. 2014; Kendal et al. 2017). At large scales, Liu et al. (2025) demonstrated that non‐native tree invasions can reduce native species richness at continental scales, primarily through the loss of locally rare native species. However, those patterns emerge in systems governed by natural regeneration, competitive interactions, and long‐term demographic replacement. By contrast, the campus landscapes examined here represent a fundamentally different assembly pathway, in which species persistence is maintained through deliberate planting rather than recruitment and competition. Our results similarly demonstrate that different habitat types within a single managed landscape can support markedly different proportions of native and introduced taxa. Because we did not quantify mowing frequency, irrigation, planting frequency, disturbance, or other management inputs, we do not attribute this pattern directly to differences in management intensity. Instead, the results identify lawns as a component of this campus landscape with comparatively high native representation and provide a basis for testing which ecological and management factors contribute to that pattern.

### 4.2 Functional and Taxonomic Differentiation and Overlap Between Native and Introduced Taxa

Native and introduced assemblages also differed in their growth‐form and taxonomic composition. Native taxa had a relatively high representation of long‐lived woody forms, whereas introduced taxa included proportionally more herbaceous perennials and shrubs. Despite these compositional differences, hierarchical clustering based on growth‐form and taxonomic attributes revealed substantial overlap between native and introduced taxa, with several clusters containing species from both groups. Thus, species origins were associated with differences in overall growth‐form composition but did not correspond to complete separation in the multivariate functional–taxonomic space represented by our dataset.

The observed overlap is notable because it indicates that introduced taxa do not constitute a uniformly distinct group based on the attributes considered here. Instead, native and introduced species share combinations of growth form, habitat association, and taxonomic characteristics. Functional similarity between native and introduced taxa has broader relevance to invasion and community‐assembly research, where introduced species may overlap substantially with functional roles represented within resident floras (Williams et al. 2015; Díaz et al. 2016). However, we interpret our result specifically as functional–taxonomic similarity or overlap, rather than direct evidence of functional convergence. Although convergence would also increase similarity, it implies a process through which taxa become more similar in response to shared ecological or evolutionary pressures; demonstrating such a process would require temporal, phylogenetic, or trait‐based evidence beyond the scope of the present survey.

Taxonomic composition added another dimension of differentiation. Introduced taxa were concentrated within a relatively limited set of species‐rich families, including Asteraceae, Lamiaceae, and Ericaceae, whereas native taxa were distributed across a broader range of families. Such taxonomic concentration is consistent with evidence that plant availability, nursery propagation, and consumer preferences produce non‐random representation of particular lineages in urban and horticultural floras (Russo et al. 2025). Nevertheless, our data do not directly measure nursery availability, historical planting frequency, or horticultural preference. We therefore interpret the family‐level pattern as evidence that the introduced component of the campus flora is taxonomically non‐random and compositionally different from the native component, rather than as direct evidence of the process that generated that pattern.

These findings are also relevant to broader invasion studies showing that functional and phylogenetic relationships between native and non‐native taxa can influence patterns of native richness and invasion outcomes (Liu et al. 2025). Functionally or phylogenetically similar native and introduced species may compete more strongly because of greater overlap in resource use, whereas greater dissimilarity may facilitate establishment by allowing introduced species to exploit ecological niches or resources less fully used by resident taxa. At the same time, environmental filtering may favor native and introduced species with similar traits under the same local conditions. Our campus‐scale study does not test these mechanisms, displacement, or competitive interactions and therefore cannot determine whether the functional similarity observed here affects native‐species vulnerability. Instead, it demonstrates that substantial similarity between native and introduced taxa can occur within a highly managed flora. Determining whether this similarity influences establishment, persistence, competition, or subsequent naturalization would require demographic or experimental evidence.

We found that introduced species were concentrated in a few families, such as Asteraceae, Lamiaceae, and Ericaceae, which are globally species‐rich and disproportionately represented in horticultural production. Together, the growth‐form, clustering, and family‐level results reveal both differentiation and overlap between native and introduced assemblages. They differ in the proportional representation of major growth forms and taxonomic groups, yet those differences do not translate into complete separation based on the attributes examined. This combination provides a more nuanced view of managed floras than treating native and introduced taxa as internally uniform ecological groups, and it is consistent with the broader recognition that urban plant assemblages reflect interacting ecological and human influences (Pickett et al. 2011; Aronson et al. 2016).

### 4.3 Managed Campuses as Case‐Study Systems for Native‐Introduced Plant Assembly

One of the most informative outcomes of this study is the relative over‐representation of native species in lawns, which are often assumed to be ecologically simplified components of managed landscapes. In contrast, our results show that the habitat, growth‐form, and taxonomic patterns characterize a campus flora in which native and introduced species occur within the same managed landscape but are unevenly distributed among habitat types and differ in compositional aspects. Introduced taxa were particularly associated with ornamental planting areas, native taxa were comparatively well represented in lawns, and the two groups showed both compositional differentiation and substantial overlap in the attributes examined. Such mixtures of ecological and human influences are characteristic of highly modified ecosystems, in which community composition cannot necessarily be understood solely from processes operating in less intensively modified environments (Hobbs et al. 2006; Pickett et al. 2011).

At the same time, several caveats should be considered. The co‐occurrence of native and introduced taxa should not be interpreted as necessarily demonstrating stable ecological coexistence, competitive displacement, or invasion success. Co‐occurrence in a floristic survey is not equivalent to demographic processes, which would require information on recruitment, survival, population growth, and interactions through time. Also, the greater representation of introduced taxa in particular habitats cannot distinguish deliberate planting from subsequent naturalization or invasion. This distinction is important because the ecological consequences of introduced plants depend strongly on context, spatial scale, disturbance history, and establishment status (USGS 2023).

These considerations also place the present findings in an appropriate context relative to studies of biological invasion in less intensively managed systems. For example, non‐native tree invasions can be associated with reductions in native species richness at broad spatial scales, particularly through losses of locally rare native taxa (Liu et al. 2025). Our campus survey addresses a different ecological context: a managed landscape in which plant composition and habitat associations are continually influenced by human activities such as planting, mowing, landscaping, and vegetation removal. We therefore do not interpret the absence of evidence for displacement in our dataset as evidence that displacement does not occur. Rather, our results describe how native and introduced taxa are currently distributed within this managed landscape and identify patterns that subsequent studies can evaluate mechanistically.

Several limitations define the scope of these inferences. The survey represents approximately 6.7 ha of a single university campus sampled for one autumn season. It documents species occurrence rather than abundance and does not quantify propagule pressure, recruitment, mortality, planting history, management intensity, or temporal changes in populations. Habitat categories consequently describe broad landscape contexts rather than quantitatively measured management gradients, and habitat‐associated differences should not be interpreted as direct effects of management intensity. These limitations constrain inference about the processes generating the observed patterns, particularly environmental filtering, competitive displacement, demographic persistence, and invasion dynamics. More broadly, ecological effects of introduced species vary substantially with spatial scale and environmental and management context (USGS 2023; Russo et al. 2025), further emphasizing the importance of interpreting our results at the spatial and temporal scale at which they were collected.

Despite these limitations, university campuses provide useful case‐study systems for documenting native and introduced floras under sustained human management. Campuses combine ornamental plantings, lawns, and other green spaces within spatially compact landscapes, creating opportunities for repeated observation and experimental investigation (Kendal et al. 2017). The patterns identified here generate specific testable hypotheses. For example, repeated floristic surveys could determine whether habitat‐associated differences in species origin remain stable through time, while distinguishing planted from self‐established individuals could clarify the contribution of spontaneous recruitment. Quantitative measurements of mowing, irrigation, disturbance, planting frequency, and other management inputs could also test whether the observed habitat associations correspond to specific management practices. Additionally, future studies could examine how social–horticultural processes, such as plant availability, esthetic preferences, and repeated planting, interact with ecological conditions, such as mowing, disturbance, and local habitat characteristics, to shape native and introduced plant assemblages. Finally, experimental planting approaches could test whether native and introduced taxa with comparable functional attributes differ in establishment or persistence under equivalent conditions.

Collectively, this study does not necessarily demonstrate a particular invasion mechanism, but establishes an empirical baseline for understanding how native and introduced taxa are organized across habitat, functional, and taxonomic dimensions within a managed campus landscape. Linking these compositional patterns with demographic, temporal, and quantitative management data would provide the next step toward determining the processes responsible for their assembly and their potential connections to naturalization and biological invasion (USGS 2023; Russo et al. 2025).

## 5 Conclusions

Our study demonstrates clear habitat‐associated, growth‐form, and taxonomic differences between native and introduced plants within a managed campus landscape. Introduced taxa were disproportionately represented in ornamental habitats, whereas native taxa were comparatively well represented in lawns. Despite these differences, substantial functional–taxonomic overlap indicates that species origin does not correspond to complete compositional separation. These patterns provide a baseline for understanding native and introduced plant assemblages in managed landscapes, although demographic, temporal, and quantitative management data are needed to identify the processes underlying them.

From a management perspective, the relatively high representation of native taxa in some campus habitats highlights opportunities to increase their use in landscape design. For example, small‐scale experimental plantings of native taxa with characteristics comparable to commonly used ornamentals could evaluate their performance under similar management conditions while potentially supporting broader ecological functions (Tallamy 2007; Burghardt et al. 2009; Perre et al. 2011; Tartaglia and Aronson 2024; Lilkendey et al. 2025; but see Matteson and Langellotto 2011). Repeated surveys, experimental plantings, and long‐term monitoring could thus establish campuses as useful systems for evaluating native plant performance and informing evidence‐based landscape management.

## Author Contributions

**Santosh K. Rana:** conceptualization (lead), data curation (equal), formal analysis (lead), investigation (equal), methodology (lead), visualization (lead), writing – original draft (lead). **Gary L. Davenport:** data curation (equal), investigation (equal), validation (equal), writing – review and editing (equal). **Serene Awad:** data curation (equal), investigation (equal), validation (equal), writing – review and editing (equal). **Donald J. Webb:** data curation (equal), investigation (equal), validation (equal), writing – review and editing (equal). **Samuel G. Esposito:** data curation (equal), investigation (equal), validation (equal), writing – review and editing (equal). **Cuong D. Nguyen:** data curation (equal), investigation (equal), validation (equal), writing – review and editing (equal). **Ian J. Renne:** investigation (equal), validation (equal), writing – review and editing (equal).

## Funding

The authors have nothing to report.

## Ethics Statement

This research involved non‐destructive observational surveys of publicly accessible plant communities and did not require ethical approval.

## Conflicts of Interest

The authors declare no conflicts of interest.

## Supporting information


**Figure S1:** Trait‐based dendrogram of native and introduced plant species across campus habitats.

**Table S1:** Representative plant species documented during the campus floristic survey. Columns indicate scientific name, growth habit, family, origin status (native/introduced), and the type of managed habitat in which each species was recorded (lawn, bed, or flowerbed).

## Data Availability

The data supporting this study are provided in Table S1. Additional analytical scripts and processed datasets are available from the corresponding author upon reasonable request.
