# Supplementary material for: Native and Introduced Plants in a Managed Campus Landscape: Habitat Associations, Functional Overlap, and Taxonomic Patterns
Source: Ecol Evol. 2026 Sep 17;16(9):e74348. doi: 10.1002/ece3.74348 (PMC13585474; doi:10.1002/ece3.74348)
Supplement: Supplementary file 1 [file ECE3-16-e74348-s001.docx]

Supplementary Figure

**Figure S1.** **Trait-based dendrogram of native and introduced plant species across campus habitats.**

Hierarchical clustering of plant species based on mixed categorical traits, including growth habit, taxonomic family, habitat type, and origin (native vs. introduced). Pairwise dissimilarities were calculated using Gower distance, which accommodates mixed data types, and species were clustered using Ward’s minimum variance method. Species labels are colored by origin (blue = native; ochre/orange = introduced). The horizontal axis represents cluster height (Gower distance), with larger values indicating greater trait dissimilarity among species. The dendrogram highlights trait-based groupings of species and the degree of overlap or separation between native and introduced taxa across managed campus environments.
