# Supplementary material for: Native and Introduced Plants in a Managed Campus Landscape: Habitat Associations, Functional Overlap, and Taxonomic Patterns
Source: Ecol Evol. 2026 Sep 17;16(9):e74348. doi: 10.1002/ece3.74348 (PMC13585474; doi:10.1002/ece3.74348)
Supplement: Supplementary file 2 [file ECE3-16-e74348-s002.docx]

**Supplementary Table**

**Table S1.** Representative native plant species documented during the campus floristic survey. Columns indicate scientific name, growth habit, family, origin status (native), and the type of managed habitat in which each species was recorded (lawn, bed, or flowerbed).

| **Scientific name** | **Habit** | **Family** | **Status** | **Location type** |
| --- | --- | --- | --- | --- |
| *Toxicodendron radicans* (L.) Kuntze | Perennial semi-woody vine | Anacardiaceae | native | lawn |
| *Asclepias syriaca* L. | Herbaceous perennial | Asclepiadaceae | native | flowerbed |
| *Ageratina altissima* (L.) R.M.King & H.Rob. | Herbaceous perennial | Asteraceae | native | lawn |
| *Packera* spp*.* | Annual or perennial herb | Asteraceae | native | lawn |
| *Echinacea purpurea* (L.) Moench | Herbaceous perennial | Asteraceae | native | flowerbed |
| *Rudbeckia hirta* L. | Herbaceous annual/short lived perennial | Asteraceae | native | bed |
| *Betula papyrifera* Marshall | deciduous tree | Betulaceae | native | lawn |
| *Catalpa speciosa* (Warder ex Barney) Engelm. | deciduous tree | Bignoniaceae | native | lawn |
| *Campsis radicans* (L.) Seem. ex Bureau | Perennial woody vine | Bignoniaceae | native | flowerbed |
| *Cornus florida* L. | Deciduous tree | Cornaceae | native | lawn |
| *Cyperus esculentus* L. | Broadleaved perennial herb | Cyperaceae | native | lawn |
| *Euphorbia maculata* L. | Herbaceous prostrate mat-forming annual | Euphorbiaceae | native | lawn |
| *Acalypha rhomboidei* Raf. | Evergreen shrub | Euphorbiaceae | native | lawn |
| *Cercis canadensis* L. | Deciduous tree | Fabaceae | native | lawn |
| *Gymnocladus dioicus* (L.) K.Koch | Deciduous tree | Fabaceae | native | lawn |
| *Gleditsia triacanthos* L. | Deciduous tree | Fabaceae | native | lawn |
| *Quercus coccinea* Münchh. | Deciduous tree | Fagaceae | native | lawn |
| *Quercus palustris* Münchh. | Deciduous tree | Fagaceae | native | lawn |
| *Quercus alba* L. | Deciduous tree | Fagaceae | native | lawn |
| *Morus rubra* L. | Deciduous tree | Moraceae | native | lawn |
| *Epilobium coloratum* Biehler | Perennial forb | Onagraceae | native | flowerbed |
| *Oxalis stricta* L. | Herbaceous annual | Oxalidaceae | native | lawn |
| *Phytolacca americana* L. | Shrub-like herbaceous perennial | Phytolaccaceae | native | lawn |
| *Pinus strobus* L. | Evergreen conifer tree | Pinaceae | native | lawn |
| *Larix laricina* (Du Roi) K.Koch | Desciduous conifer tree | Pinaceae | native | lawn |
| *Platanus occidentalis* L. | Deciduous tree | Platanaceae | native | lawn |
| *Polemonium reptans* L. | Herbaceous perennial | Polemoniaceae | native | bed |
| *Crataegus* spp*.* | Deciduous tree | Rosaceae | native | bed |
| *Amelanchier* sp*.* | Deciduous shrub | Rosaceae | native | bed |
| *Aesculus glabra* Wild. | Deciduous tree | Sapindaceae | native | lawn |
| *Acer pensylvanicum* L. | Deciduous tree | Sapindaceae | native | lawn |
| *Typha* spp*.* | Herbaceous wetland perennial | Typhaceae | native | flowerbed |
| *Ulmus japonica × wilsoniana* | Deciduous tree | Ulmaceae | native | lawn |
| *Ulmus rubra* Muhlenb. | Deciduous tree | Ulmaceae | native | lawn |
| *Boehmeria cylindrica* (L.) Sw. | Herbaceous perennial | Urticaceae | native | lawn |
| *Viburnum lantana* L. | Deciduous shrub | Adoxaceae | introduced | lawn |
| *Liquidambar formosana* Hance | Deciduous tree | Altingiaceae | introduced | lawn |
| *Cotinus coggygria* Scop. | Perennial shrub | Anacardiaceae | introduced | lawn |
| *Vinca minor* L. | Herbaceous perennial | Apocynaceae | introduced | flowerbed |
| *Caladium bicolor* (Aiton) Vent. | Tuburous perennial | Araceae | introduced | flowerbed |
| *Colocasia esculenta* (L.) Schott | Herbaceous perennial | Araceae | introduced | flowerbed |
| *Hedera helix* L. | Evergreen vine | Araliaceae | introduced | lawn |
| *Hosta plantaginea* (Lam.) Asch. | Herbaceous perennial | Asparagaceae | introduced | flowerbed |
| *Liriope muscari* (Decne.) L.H.Bailey | Herbaceous perennial | Asparagaceae | introduced | lawn |
| *Hemerocallis fulva* (L.) L. | Herbaceous perennial | Asphodelaceae | introduced | bed |
| *Tagetes erecta* L. | Herbaceous annual | Asteraceae | introduced | bed |
| *Cirsium arvense* (L.) Scop. | Herbaceous perennial | Asteraceae | introduced | flowerbed |
| *Taraxacum officinale* F.H.Wigg. | Herbaceous perennial | Asteraceae | introduced | lawn |
| *Coreopsis verticillata* L. | Herbaceous bushy perennial | Asteraceae | introduced | lawn |
| *Leucanthemum vulgare* Lam. | Herbaceous perennial | Asteraceae | introduced | flowerbed |
| *Sonchus oleraceus* L. | Herbaceous annual | Asteraceae | introduced | flowerbed |
| *Sonchus asper* (L.) Hill | Herbaceous annual | Asteraceae | introduced | flowerbed |
| *Berberis thunbergii* DC. | Deciduous shrub | Berberidaceae | introduced | lawn |
| *Cardamine hirsute* L. | Herbaceous annual | Brassicaceae | introduced | lawn |
| *Pachysandra terminalis* Siebold & Zucc. | Evergreen herbaceous perennial | Buxaceae | introduced | flowerbed |
| *Canna indica* L. | Herbaceous perennial | Cannaceae | introduced | flowerbed |
| *Stellaria media* (L.) Vill. | Herbaceous mat-forming winter annual | Caryophyllaceae | introduced | lawn |
| *Euonymus alatus* (Thunb.) Siebold | Deciduous woody perennial shrub | Celastraceae | introduced | lawn |
| *Commelina communis* L. | Herbaceous annual | Commelinaceae | introduced | flowerbed |
| *Cornus kousa* F.Buerger ex Miq. | Deciduous tree | Cornaceae | introduced | flowerbed |
| *Sedum sarmentosum* Bunge | Mat-forming perennial succulent groundcover | Crassulaceae | introduced | bed |
| *Juniperus rigida* Siebold & Zucc. | Evergreen tree | Cupressaceae | introduced | bed |
| *Cyperus* spp*.* | Perennial broadleaved herb | Cyperaceae | introduced | lawn |
| *Rhododendron catawbiense* Michx. | Evergreen shrub | Ericaceae | introduced | bed |
| *Rhododendron* hybrid | Shrub | Ericaceae | introduced | flowerbed |
| *Rhododendron indicum* (L.) Sweet | Evergreen shrub | Ericaceae | introduced | flowerbed |
| *Rhododendron ponticum* L. | Evergreen shrub | Ericaceae | introduced | flowerbed |
| *Rhododendron* spp*.* | Shrub | Ericaceae | introduced | flowerbed |
| *Gleditsia triacanthos* L. | deciduous tree | Fabaceae | introduced | flowerbed |
| *Trifolium repens* L. | Herbaceous perennial | Fabaceae | introduced | lawn |
| *Castanea mollissima* Blume | Deciduous tree | Fagaceae | introduced | lawn |
| *Quercus shumardii* Buckley | Deciduous tree | Fagaceae | introduced | lawn |
| *Ginkgo biloba* L. | deciduous tree | Ginkgoaceae | introduced | lawn |
| *Fothergilla gardenii* L. | Deciduous shrub | Hamamelidaceae | introduced | flowerbed |
| *Hydrangea paniculata* Siebold | Deciduous shrub | Hydrangeaceae | introduced | flowerbed |
| *Hydrangea* sp*.* | Deciduous shrub | Hydrangeaceae | introduced | flowerbed |
| *Salvia yangii* B.T.Drew | Herbaceous perennial | Lamiaceae | introduced | lawn |
| *Glechoma hederacea* L. | Herbaceous creeping perennial | Lamiaceae | introduced | lawn |
| *Lamium purpureum* L. | Herbaceous annual | Lamiaceae | introduced | lawn |
| *Nepeta* spp*.* | Herbaceous perennial | Lamiaceae | introduced | bed |
| *Salvia nemorosa* L. | Evergreen shrub | Lamiaceae | introduced | flowerbed |
| *Salvia* sp*.* | Perennial shrub | Lamiaceae | introduced | flowerbed |
| *Solenostemon scutellarioides* (L.) Codd | Herbaceous perennial | Lamiaceae | introduced | bed |
| *Magnolia Kobus* DC. | Deciduous shrub | Magnoliaceae | introduced | flowerbed |
| *Magnolia virginiana* L. | Deciduous shrub | Magnoliaceae | introduced | bed |
| *Hibiscus grandifloras* Michx. | Herbaceous perennial | Malvaceae | introduced | flowerbed |
| *Tilia tomentosa* Moench | Deciduous tree | Malvaceae | introduced | lawn |
| *Tilia cordata* Mill. | Deciduous tree | Malvaceae | introduced | lawn |
| *Ligustrum obtusifolium* Siebold & Zucc. | Descidous Semi-evergreen shrub | Oleaceae | introduced | lawn |
| *Paeonia lactiflora* Pall. | Herbaceous perennial | Paeoniaceae | introduced | bed |
| *Pinus resinosa* Aiton | Coniferous evergreen tree | Pinaceae | introduced | lawn |
| *Veronica arvensis* L. | Herbaceous winter annual | Plantaginaceae | introduced | bed |
| *Plantago major* L. | Herbaceous perennial | Plantaginaceae | introduced | lawn |
| *Linaria vulgaris* Mill. | Herbaceous perennial | Plantaginaceae | introduced | lawn |
| *Miscanthus sinensis* Anderson | Perennial broadleaved herb | Poaceae | introduced | bed |
| *Digitaria sanguinalis* (L.) Scop. | Annual broadleaved herb | Poaceae | introduced | lawn |
| *Fallopia scandens* (L.) Holub | Perennial vine | Polygonaceae | introduced | flowerbed |
| *Portulaca oleracea* L. | Annual succulent | Portulacaceae | introduced | bed |
| *Aquilegia vulgaris* L. | Herbaceous perennial | Ranunculaceae | introduced | flowerbed |
| *Rhamnus cathartica* L. | Deciduous shrub | Rhamnaceae | introduced | lawn |
| *Pyrus calleryana* Decne. | Deciduous tree | Rosaceae | introduced | lawn |
| *Malus* spp*.* | Deciduous tree | Rosaceae | introduced | lawn |
| *Duchesnea indica* (Jacks.) Focke | Herbaceous perennial | Rosaceae | introduced | lawn |
| *Galium aparine* L. | Herbaceous broadleaved annual | Rubiaceae | introduced | flowerbed |
| *Acer palmatum* Thunb. | Deciduous tree | Sapindaceae | introduced | lawn |
| *Acer platanoides* L. | Deciduous tree | Sapindaceae | introduced | lawn |
| *Ailanthus altissima* (Mill.) Swingle | Deciduous tree | Simaroubaceae | introduced | lawn |
| *Solanum dulcamara* L. | Perennial semi-woody vine | Solanaceae | introduced | bed |
| *Staphylea pinnata* L. | Deciduous shrub | Staphyleaceae | introduced | flowerbed |
| *Taxus cuspidate* Siebold & Zucc. | Perennial everegreen shrub | Taxaceae | introduced | flowerbed |
| *Glandularia guaranitica* (A.St.-Hil.) P.W.Michael | Herbaceous perennial | Verbanaceae | introduced | flowerbed |
| *Glandularia* spp. | Herbaceous perennial | Verbanaceae | introduced | flowerbed |
